# Ginsenoside Rg3 Sensitizes Colorectal Cancer to Radiotherapy through Downregulation of Proliferative and Angiogenic Biomarkers

**DOI:** 10.1155/2018/1580427

**Published:** 2018-03-18

**Authors:** Taiguo Liu, Lina Duo, Ping Duan

**Affiliations:** ^1^Department of Medical Oncology, Chengdu Integrated TCM & Western Medicine Hospital, Chengdu First People's Hospital, Chengdu, Sichuan, China; ^2^Department of Dermatology, Chengdu Integrated TCM & Western Medicine Hospital, Chengdu First People's Hospital, Chengdu, Sichuan, China

## Abstract

**Background:**

Radiation therapy is an important mode of colorectal cancer treatment. However, most people die of local recurrence after tumors become resistant to radiotherapy, and little progress has been made in treating radiotherapy-resistant colorectal cancer. Hence, novel agents that are nontoxic and can sensitize colorectal cancer to radiotherapy are urgently needed. Ginsenoside Rg3, a saponin extracted from ginseng, shows cytotoxicity against a variety of cancer cells through suppression of pathways linked to oncogenesis, including cell survival, proliferation, invasion, and angiogenesis. In this article, we investigated whether Rg3 can sensitize colorectal cancer to radiation in vivo.

**Methods and Materials:**

We established CT-26 xenografts in BALB/c mice and treated them with vehicle, Rg3, radiation, and combined Rg3 + radiation. Mouse quality of life, survival, tumor volumes, and inhibitive rates were estimated. NF-*κ*B activation was ascertained using electrophoretic mobility shift assay and immunohistochemistry. We also tested for markers of proliferation, angiogenesis, and invasion using immunohistochemistry and Western blot analysis.

**Results:**

Rg3 significantly enhanced the efficacy of fractionated radiotherapy by improving the quality of life of mice. Moreover, tumors from mice xenografted with CT-26 cells and treated with combined Rg3 + radiotherapy showed significantly lower tumor volumes (*P* < 0.01 versus controls; *P* < 0.05 versus radiation alone), NF-*κ*B activation, and expression of NF-*κ*B-regulated gene products (cyclin D1, survivin, cyclooxygenase-2 (COX-2), and vascular endothelial growth factor (VEGF)) compared with controls. The combination treatment was also effective in suppressing angiogenesis, as indicated by lower CD31^+^ microvessel density compared with controls (*P* < 0.05).

**Conclusion:**

Our results suggest that Rg3 enhances the antitumor effects of radiotherapy for colorectal cancer by suppressing NF-*κ*B and NF-*κ*B-regulated gene products, leading to inhibition of tumors and prolongation of the lifespan of CT-26 xenograft BALB/c mice.

## 1. Introduction

Colorectal cancer is the third most common type of cancer in western countries and is a significant cause of morbidity and mortality throughout the world [1]. Neoadjuvant chemoradiation therapy (CRT) is widely used to decrease local recurrence after surgical resection because colorectal cancer frequently presents at a locally advanced stage [2]. It can also improve disease-free and overall survival rates and increases the chances of anal sphincter preservation at the time of surgery [3, 4]. Unfortunately, more than one-third of patients do not respond or show only modest responses to CRT and optimal surgery treatment [5]. Furthermore, complete pathologic response to neoadjuvant CRT assessed at the time of surgical resection has become a surrogate marker for outcome. However, only about 10–20% of patients achieve a complete pathologic response to neoadjuvant CRT due to their resistance to radiation therapy [6, 7]. Developing a strategy to improve local control and pathologic response rates with novel radiosensitization may keep some patients from radical surgical resections.

It is not clear why the response to radiation is so limited. Recently, most reported studies have shown that the transcription factor NF-*κ*B plays a critical role in the development of radiotherapy suppression on tumor [8]. NF-*κ*B has been found to mediate tumor promotion, metastasis, angiogenesis, and treatment resistance in a wide range of tumors through the expression of genes involving in malignant transformation and tumor promotion [9, 10]. Previous study showed that NF-*κ*B is constitutively active in colon cancer cells, but not in normal colorectal ductal epithelial cells [11]. Several studies have suggested that radiotherapy induces NF-*κ*B activity upregulation [12–14]. Therefore, agents that inhibit NF-*κ*B activity could potentiate the radioresponse in colorectal cancer.

*Panax ginseng* has been frequently used to treat many disorders in traditional Chinese medicine for more than 2000 years. The principle biologically active ingredient of ginseng is ginsenoside, which has been found to have many biological activities, including anti-inflammatory and antineoplastic effects [15]. Ginsenoside Rg3 extracted from* P. ginseng* has been shown to inhibit a variety of tumors [16–18].

It is not yet known whether Rg3 can sensitize colorectal tumors to radiotherapy. So, in this study, we measured the effect of Rg3 on the growth of colorectal cancer exposed to radiation, emphasizing combination therapy and inhibition of NF-*κ*B as a molecular mechanism.

## 2. Methods and Materials

### 2.1. Materials

Ginsenoside Rg3 extracted from ginseng was supplied by the YaTai Pharmaceutical Company (China) with a purity of above 99.5%. Immunohistochemistry was performed by Liquid DAB+ substrate-chromogen system (Dako). Polyclonal antibodies against p65, cyclin D1, and survivin and monoclonal antibodies against VEGF, CD31, COX-2, and *β*-actin were purchased from Santa Cruz Biotechnology. Electrophoretic mobility shift assay (EMSA) kit was obtained from Promega. All other chemicals were purchased from the Beyotime Institute of Biotechnology (China), unless otherwise indicated.

### 2.2. Cell Culture

The CT-26 murine colon carcinoma cell line was supplied by the Cancer Research Institution at Sichuan University. The CT-26 cells were routinely cultured in RPMI1640 medium containing 10% fetal bovine serum (Gibco) with 100 U/mL penicillin and 100 *μ*g/mL streptomycin and kept at 37°C in a humidified atmosphere of 5% CO_2_.

### 2.3. Animals and Experimental Protocol

Male BALB/c mice (aged 4 weeks) were used for experiments, which were obtained from the Experimental Animal Center of the West China School of Medicine, Sichuan University. The animals were housed (four mice per cage) under standard conditions (20–26 °C, 40–70% humidity, a 12-hour light/dark cycle) and were given food and water (ad libitum). None of the mice exhibited any lesions and all tested as pathogen-free. Cultured CT-26 colon cancer cells were washed with phosphate-buffered saline (PBS) and resuspended in a serum-free medium. The mouse was subcutaneously injected with the suspension (5 × 10^6^ cells in 0.2 ml) under the armpits of the right anterior superior limbs. Seven days later, when the tumors were palpable, the mice were randomly divided into the following four groups (10 mice per group): (1) a control group receiving intragastric normal saline administration for 30 days, (2) a Rg3 group receiving intragastric administration of Rg3 (10 mg/kg) daily for 30 days, (3) a radiation group, which received radiotherapy delivered directly to the site of tumor twice weekly (2 Gy) for 2 weeks, and (4) a combination group (Rg3 + radiotherapy) treated with Rg3 (10 mg/kg) daily for 30 days and radiation delivered to the site of tumor twice weekly (2 Gy) for 2 weeks. For radiation treatment, mice were first anaesthetized with a volume of 70–100 *μ*l mixture of ketamine (20 mg/ml) and xylazine (20 mg/ml) at a ratio of 6.7 : 1 and then placed onto a specially designed lead plate so as to radiate locally the tumors. The animals were euthanatized six weeks after tumor cell injection and tumor samples were immediately removed. All animal procedures were approved and performed in accordance with the guidelines of the Animal Care and Scientific Committee of Chengdu Integrated TCM & Western Medicine Hospital.

### 2.4. Electrophoretic Mobility Shift Assays (EMSA)

EMSA was performed to assess NF-*κ*B activation. Briefly, cell nucleus extracted from tumor samples was incubated with ^32^P-labeled NF-*κ*B consensus oligonucleotide for 20 min at room temperature. The resulting DNA-protein complex was separated from the free oligonucleotides on a nondenaturing 4% polyacrylamide gel and then visualized by the Imaging System.

### 2.5. Immunolocalization of NF-KB p65 in Tumor Samples

The nuclear localization and expression of p65 in cancer tissues and adjacent tissues were determined by immunohistochemical method. Briefly, colorectal tumor samples were fixed in 4% paraformaldehyde, embedded in paraffin, and sectioned. The biopsies were washed with PBS, blocked for 20 min in protein block solution, and incubated by the antibody to p65 (1 : 100). Biotinylated secondary antibodies were used and stained by Liquid DAB+. Finally, sections were counterstained with hematoxylin and microscopically analyzed with a FV1000 Laser Scanning Confocal Microscope (OLYMPUS).

### 2.6. Detection of Microvessel Density

Microvessel density (MVD) was assessed and determined by immunohistochemical examination using antibody to the endothelial marker CD31. Sections were fixed, embedded, and stained with rat anti-mouse CD31 monoclonal antibody (1 : 50). Areas of highest vessel density were then assessed under high magnification and capillaries and arterioles were counted. The average vessel count in three hot spots was blindly measured by three investigators and used as the MVD value. The consensus of microvessel counts was considered as the final score for analysis.

### 2.7. Western Blot Analysis

Colorectal tumor tissues (75–100 mg/mouse) from control and experimental mice were homogenized with 0.5 mL of ice-cold RIPA lysis buffer containing a proteinase inhibitor mixture (Roche) on ice for 30 min. The homogenate was rotated at 4°C for 20 min and centrifuged at 12,000/g at 4°C for 15 min and the supernatant was collected. Crude protein extracts (40 *μ*g), the concentration of which were measured by BCA method, were denatured and fractionated by SDS-PAGE, electrotransferred onto nitrocellulose membranes (Millipore). The membranes were blotted in TBST (25 mM Tris, pH 7.4, 137 mM NaCl, 2.7 mM KCl, and 0.05% Tween 20) containing 5% nonfat milk or 5% BSA for 1 hour at room temperature and visualized using antibody against VEGF, COX-2, cyclin D1, survivin, and *β*-actin (1 : 1000, 1 : 500, 1 : 500, 1 : 500, and 1 : 2000, resp.) and detected by an enhanced chemiluminescence (ECL) detection system (Pierce). The density of the selected bands was quantified using ImageJ software.

### 2.8. Statistical Analysis

**Figure 1:**
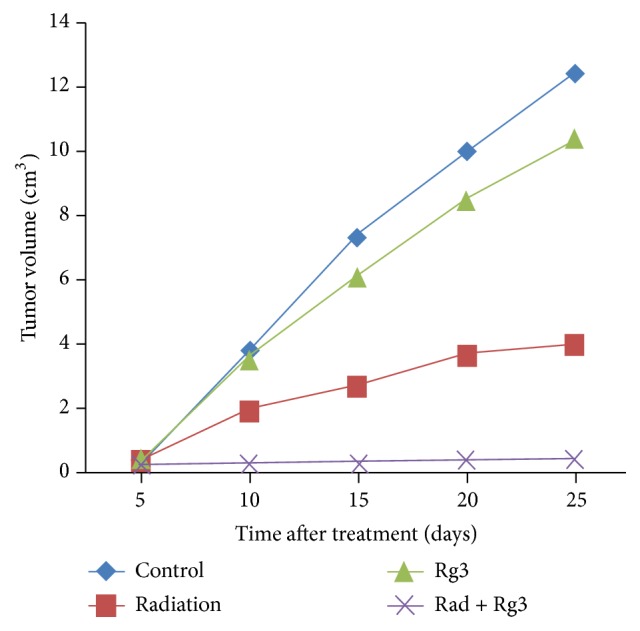
Tumor growth over time in the different treatment groups of mice. (Blue) controls, (red) radiation-treated, (green) Rg3-treated, and (purple) combined Rg3 and radiation-treated mice. All data are expressed as mean.

Data were analyzed using SPSS 19.0 software. Results were given as mean ± SEM, except for Figure 1 whose data are showed as mean only. *P* < 0.05 was considered statistically significant. Two-way ANOVA followed by Bonferroni posttest was used to compare different groups. For all other statistical analyses a two-tailed unpaired *t*-test was used.

## 3. Results

The goal of this study was, first, to determine whether Rg3 potentiates the antitumor effects of radiotherapy in vivo against colorectal cancer and, second, to delineate the mechanism by which Rg3 mediates its effects.

### 3.1. Rg3 Potentiates the Antitumor Effects of Radiation in Colorectal Cancers in Xenografted Mice

Based on tumor volume measurements on the seventh day after tumor cell implantation, we randomized animals into four groups and treated them as described in* Animals and Experimental Protocol*. The animals were sacrificed six weeks after tumor cell injection, five weeks from the date of treatment. Three mice from each of the groups were sacrificed on the 10th day and analyzed for NF-*κ*B and other biomarkers. The lengths and widths of tumors were measured by caliper every five days to monitor tumor growth, and tumor volume (TV) was estimated using the formula: TV (mm^3^) = (width^2^  ×  length)/2. As shown in Figure 1, the tumor volume in the combined Rg3-radiation group was significantly lower than those of the other three groups by day 25 of treatment (*P* < 0.05 versus radiation; *P* < 0.05 versus Rg3; *P* < 0.01 versus control).

### 3.2. Rg3 Inhibits NF-*κ*B Activation in Colorectal Cancers in Xenografted Mice

**Figure 2:**
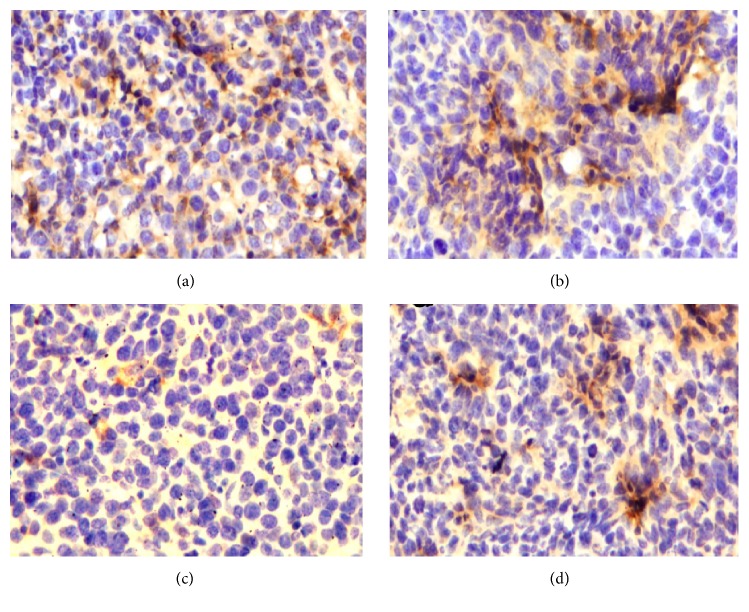
Immunohistochemical analysis of nuclear p65, showing the inhibition of NF-*κ*B in (a) controls, (b) radiation-treated, (c) Rg3-treated, and (d) radiation and Rg3-treated mice.

**Figure 3:**
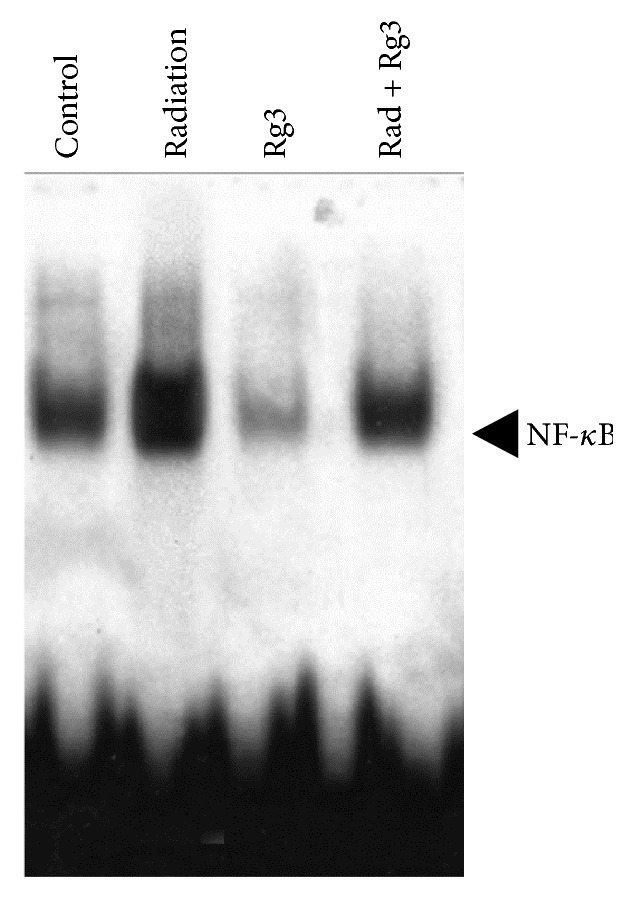
Detection of NF-*κ*B by DNA binding in colorectal tumor tissue samples showing the inhibition of NF-*κ*B by Rg3.

NF-*κ*B contributes to the development and/or progression of malignancy by regulating the expression of genes involved in cell growth and proliferation, antiapoptosis, angiogenesis, and metastasis. Whether Rg3 can affect NF-*κ*B p65 expression in vivo was first examined by immunohistochemical analysis. As shown in Figure 2, radiation activated NF-*κ*B in tumor tissues; however NF-*κ*B was inhibited by Rg3 alone or in combination with radiation. In addition, we also analyzed the effect of Rg3 and radiation on NF-*κ*B activation in tumor tissues by EMSA, and the results showed that Rg3 downregulated NF-*κ*B in tumor tissue (see Figure 3).

### 3.3. Rg3 Downregulates the NF-*κ*B-Regulated Gene Products in Tumor Tissues

**Figure 4:**
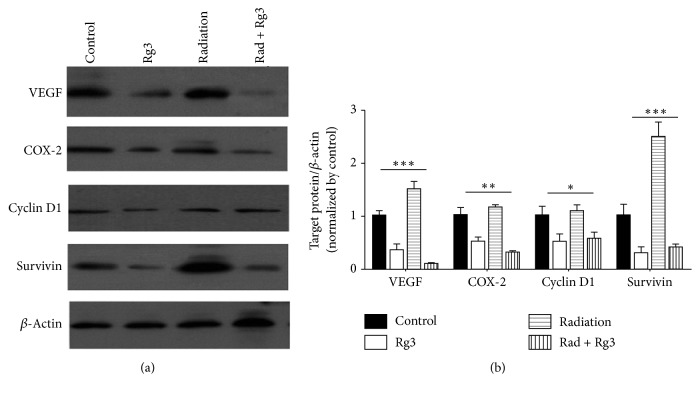
Rg3 downregulates the expression of NF-*κ*B-regulated gene products in colorectal tumor samples. Western blots showing that combined Rg3 and radiation treatment inhibits the expression of the NF-*κ*B-dependent antiapoptotic gene products survivin; angiogenic gene product VEGF; and proliferative gene products such as COX-2 and cyclin D1. (a) Representative blot, (b) the quantitative analysis for the expression of NF-*κ*B-regulated gene products. ^*∗*^*P* < 0.05, ^*∗∗*^*P* < 0.01, and ^*∗∗∗*^*P* < 0.001. Data represent mean ± SEM. All above experiments were repeated at least four times.

Because NF-*κ*B regulates the expression of the antiapoptotic and proliferative proteins such as cyclin D1, COX-2, VEGF, and survivin, we tested whether Rg3 can modulate the expression of these gene products in tumor tissues in xenografts. As shown in Figure 4, Western blot revealed significant reductions in the expressions of these gene products in tumors of the Rg3-alone or radiation-combined treatment groups compared to controls.

### 3.4. Rg3 Potentiates the Effect of Radiation on a Biomarker of Angiogenesis

**Figure 5:**
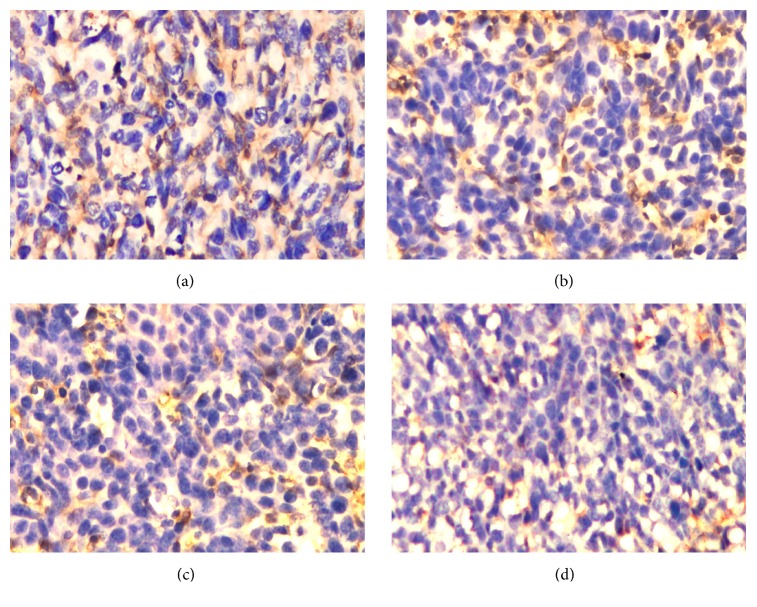
Immunohistochemical analysis of CD31 for microvessel density in colorectal tumors indications. (a) Controls, (b) radiation-treated, (c) Rg3-treated, and (d) radiation and Rg3-treated mice.

Antiangiogenesis can normalize the microvasculature of tumors, improve their lack of oxygen, and sensitize tumors to radiation. Our previous experiment showed that combining Rg3 with radiation can inhibit angiogenesis of lung cancer in mice [17]. We thus analyzed the effect of Rg3 on angiogenesis by examining the expression of CD31, which is a marker of microvessel density. The result showed that the combination of radiation and Rg3 treatments significantly suppressed the expression of CD31 in tumor tissues compared with radiation alone (see Figure 5).

### 3.5. Side Effects and Quality of Life of Mice

There were no significant abnormalities in psychosis, the status of activity, reaction to stimulation, loss of weight, appetite, or depilation of mice in the ginsenoside Rg3 group; however, this was not the case in the control, radiation, or combination groups. The quality of life of mice in the ginsenoside Rg3 group was the best, while the worst was found in the radiation group. The side effects were lower and quality of life better in the combination group compared with the gemcitabine group. The results showed that ginsenoside Rg3 might potently decrease the side effects of therapy and improve quality of life in tumor-bearing mice.

## 4. Discussion

Although radiotherapy has been frequently used to treat a variety of tumors, its success was limited by acquired resistance, which has posed a significant clinical challenge. Cellular resistance can be overcome by dose escalation, but it may result in severe cytotoxicity since radiotherapy has no choice except to damage normal tissue cells and tumor cells. Chemoradiation therapy can enhance the efficacy of radiotherapy, but it will increase toxicity, and some patients may even have to stop treatment [19]. Thus, researchers are increasingly taking an interest in exploring some nontoxic agents to increase efficacy for radiation-resistant cancer cells. The present study indicates that Rg3 sensitizes colorectal tumors to radiation therapy by inactivation of NF-*κ*B in vivo. Moreover, Rg3 is a relatively safe medicine and can improve quality of life for mice with tumors.

NF-*κ*B is found to be overexpressed in a variety of solid tumors implicating its aggressive characteristic, and some studies demonstrated that NF-*κ*B increases apoptotic resistance and resistance to radiation treatment [20, 21]. NF-*κ*B can be activated by radiation in both in vitro and in vivo models and then bind to specific DNA sequences in target genes to regulate the transcription of downstream genes, which involve in immunoregulation, growth regulation, inflammation, carcinogenesis, and cell survival [22]. Among these genes, some target genes containing NF-*κ*B binding sites in their promotors, such as COX-2, cyclin D1, and survivin, have been shown to contribute radioresistance in various types of tumor cells [23–25]. Therefore, the combination of radiation and an NF-*κ*B inhibitor maybe a promising therapeutic strategy for tumor treatment. The majority of studies have demonstrated that inhibition of NF-*κ*B activation increases radiation-induced apoptosis and enhances radiosensitivity in various tumor cells, including colorectal and cervical and melanoma [26–28]. Recent reports have identified that a number of plants sensitize cancer cells to radiation. For example, curcumin has been found to modulate the radiation sensitivity of colorectal cancer cells through inhibiting constitutive and inducible NF-*κ*B activity [29, 30].

Rg3 is an effective chemical trace component of ginseng with a C42H72O13 framework and 784 Da molecular weight, and it has pleiotropic capabilities, including antitumor effect. Previous studies have reported that Rg3 inhibits tumor cells to grow, invade, metastasize, and neovascularize. In another experiment, Kim et al. reported that Rg3 enhances the susceptibility of prostate and colon cancer cells to docetaxel via inhibition of NF-*κ*B in vitro [18, 31]. Consistent with previous reports, our results confirmed that Rg3 enhances the antitumor effects of radiotherapy in CT-26 xenograft BALB/c mice by suppressing NF-*κ*B activation and key downstream tumorigenic and angiogenic factors. In addition, we found that Rg3 suppresses angiogenesis, indicating its indirect (via angiogenic mechanisms) antitumor effect.

COX-2 has also been reported to mediate radiation resistance in various tumor cells, such as laryngeal tumors, oral squamous cell carcinomas, and glioblastoma multiforme cells [32–34]. Cyclin D1 that controls the transition from G1 to S phase in the cell cycle is overexpressed in a wide variety of tumors and contributes chemoresistance and radioresistance [35–37]. Survivin as a member of the family of apoptosis protein inhibitors is a key regulator of mitosis and programmed cell death [38]. Survivin overexpression has been found to be a predictive factor of response to chemotherapy and radiotherapy in patients with a wide variety of tumors [39–41]. In our study, Western blot showed that Rg3 reduces the expression of NF-*κ*B-regulated gene products (cyclin D1, survivin, and COX2) in cancer cells, indicating that Rg3 enhances radiosensitivity of colorectal cancer.

As the creation of new blood vessel supplies the metabolic needs of rapidly proliferating malignant cells, angiogenesis is a key step for tumor growth and metastasis. VEGF is one of the most critical proangiogenic growth factors and plays an important role in tumor angiogenesis. Decreased expression of VEGF may enhance tumor cells oxygenation through normalization of aberrant vessel. It has been reported that Rg3 blocks tumor growth by targeting VEGF [42]. We measured VEGF and MVD and observed that levels of these two markers were decreased by Rg3. The results suggest that Rg3 sensitizes colorectal cancer to radiotherapy, partly through the inhibition of angiogenesis.

Although Rg3 is considered a natural product, its safety and toxicity must be addressed. Consistent with previous reports, our results confirmed that ginsenoside Rg3 not only has no toxic side effects on the marrow, heart, lung, liver, kidney, and nervous system but can also enhances the living quality of mice with tumors.

In conclusion, our results show that Rg3 potentiates the antitumor effects of radiation by inhibiting NF-*κ*B activation and its downstream targets. In addition, Rg3 involves suppression of proliferation and angiogenesis, suggesting that the underlying mechanisms seem to be multifaceted. Third, Rg3 provides a favorable safety profile and supports further research to explore it as a chemopreventive agent.
